# Evidence of rotavirus vaccine impact in sub-Saharan Africa: Systematic review and meta-analysis

**DOI:** 10.1371/journal.pone.0232113

**Published:** 2020-04-27

**Authors:** Opolot Godfrey, Weidong Zhang, Cecilia Amponsem-Boateng, Timothy Bonney Oppong, QingLin Zhao, Dankang Li

**Affiliations:** Department of Epidemiology and Biostatistics, College of Public Health, Zhengzhou University, Zhengzhou, Henan, PR China; Public Health England, UNITED KINGDOM

## Abstract

**Background:**

Over 34 countries in Africa have introduced rotavirus vaccine to their national immunization programs: monovalent (Rotarix^®^, RV1) and pentavalent (RotaTeq^®^, RV5) after South Africa introduced it in 2009. Since then several studies assessing the impact of the vaccine have been conducted. The principal aim of this study was to evaluate the impact of rotavirus vaccine in sub-Saharan Africa.

**Methods:**

A Literature search was performed using Mendeley, PubMed, ScienceDirect, grey literature and Web of Science databases of published studies from January 1, 2017, as years of recent publications on rotavirus vaccine impact in sub-Saharan Africa. A meta-analysis was conducted for rotavirus infection in children under 5 years using proportions of pre and post-vaccine introduction in these populations. Random-effect estimates were considered since the samples were from universal populations.

**Results:**

Out of the 935 articles identified, 17 studies met the inclusion for systematic review and meta-analysis. The pooled proportion for pre-vaccination period was 42%, 95% (CI: 38–46%), and reduced to 21%, 95% (CI: 17–25%) during post-vaccination period. Rotavirus diarrhea significantly reduced in children < 12 months as compared to children 12–24 months old. Seasonal peaks of rotavirus diarrhea were between June–September. However, data is limited to one year of post-vaccine introduction, and bias may present due to early vaccine impact.

**Conclusion:**

We observed that the introduction of the rotavirus vaccine was partly responsible for the significant reduction in the burden of rotavirus-associated diarrhea in sub-Saharan Africa. Therefore, there is a need to encourage the remaining countries to introduce the vaccine to their routine national immunization programs.

## Introduction

Globally rotavirus is a common cause of severe diarrhea in children less than 5 years old, [1]. Morbidity and mortality rates of children under the age of five years in many countries are greatly attributed to diarrheal diseases resulting from rotavirus infection [2]. In developing countries, particularly in sub-Saharan Africa, the majority of children get infected before 18 months of age [2], contributing to 121,000 out of the 215,000 worldwide childhood deaths from rotavirus disease [3]. Half of the rotavirus deaths in the world is accounted for by five countries, including Nigeria, Angola and the Democratic Republic of the Congo (DRC) from sub-Saharan Africa, and India and Pakistan from South Asia [3]. In Uganda, in-patient admission for one episode of severe rotavirus diarrhea costs 10% of the average family’s monthly income [4].

Improved standards in water, hygiene and sanitation do not guarantee prevention of the transmission, simply because it is extremely infectious and resilient [5]. In developing countries, malnutrition and co-infection with multiple enteric pathogens are quite frequent which further delays recovery, hinders effective treatment, and leads to further complications, like delays in growth and development of infants [6]. Therefore, the international priority to prevent and control the disease is through immunization [5,6].

The World Health Organization has prequalified four live, oral attenuated rotavirus vaccines to be used internationally, and these include; RotaSiil^™^ (bovine-human reassortant with human G1, G2, G3, and G4 bovine UK G6P; RotaTeq^™^ (reassorted bovine-human rotavirus); Rotavac^™^ (naturally occurring bovine-human reassortant neonatal G9P, also called 116E); and Rotarix^™^ (derived from a single common strain of human rotavirus) [1]. These vaccines are considered effective in preventing severe gastrointestinal diseases [7]. The World Health Organization recommends that rotavirus vaccines be introduced into every country’s national immunization program, particularly in countries where diarrheal diseases are still a major public health problem [1]. Rotavirus-related hospitalizations among children have also shown a decline and also demonstrated herd immunity among adults who are above the age bracket to receive the vaccine [8,9].

South Africa was the first African country to introduce rotavirus vaccine in 2009 [10]. Currently, it has now been introduced in the region, with additional introduction being planned. Majorly, two vaccines: monovalent (Rotarix^®^, RV1) and pentavalent (RotaTeq^®^, RV5) are being used in the region's national immunization program [11]. In sub-Saharan Africa, the highest mortality rates associated with rotavirus are in children younger than 5 years [12]. Currently, more than 70% of Gavi-eligible countries in sub-Saharan Africa have introduced rotavirus vaccines. But since the burden of rotavirus disease is still so high in this region, the remaining countries need to introduce vaccines to protect their children from rotavirus infections [12]. If rotavirus vaccines are used in all Gavi-eligible countries, the vaccine could prevent an estimated 180,000 deaths and avert 6 million clinics and hospital visits each year, thereby, saving US$68 million annually in treatment costs [13]. This study aims to evaluate the impact of the introduction of the rotavirus vaccine immunization programs against all-cause and rotavirus specific diarrhea among children under five years in sub-Saharan Africa.

## Materials and methods

### Literature search strategy

A systematic search was performed using PubMed, ScienceDirect, grey literature, WHO, Gavi and Web of Science databases of current studies published from January 1, 2017. Based on our review of papers, the rotavirus vaccine was introduced in the majority of African countries' national immunization program from 2012 onwards, and most studies from those countries evaluating the impact were published from 2017. Since there were already impact studies conducted for the countries that introduced the rotavirus vaccine before 2012. We decided to undertake a comprehensive literature search using an African search filter, to identify rotavirus vaccine impact studies from countries that introduced the vaccine in 2012 and above (S1 File). Full articles were identified by a defined search strategy and were considered for inclusion by predetermined criteria. To ensure that records indexed using regional terms rather than country-specific terms, African filter comprised African country names as well as shortened terms such as ‘East* Africa’. Both English and other relevant languages were included in the search for African country names. (MeSH in PUBMED / MEDLINE and EMTREE in EMBASE) were combined in the database subject headings.

### Selection criteria (inclusion and exclusion)

Studies whose full text was available in English and relevant languages were eligible for inclusion. Such as all observational studies based on hospital surveillance, that: (1) defined rotavirus-positive diarrhea samples, as > 3 semi-liquid or liquid stools in 24 hours and for the duration of < 7 days, (2) samples originating from children aged 28 days to 5 years of age, (3) rotavirus genotype data from >20 samples using either ELISA for genotyping, polyacrylamide or RT-PCR laboratory techniques and also studies that provided pre and post-vaccination data. We excluded papers that did not show the number of patients with rotavirus infection during the pre and post-vaccination period or percentages that could be used for calculating the difference in proportions. Lastly, we excluded studies conducted in North Africa, because our aim was the impact of the vaccine in sub-Saharan African countries.

### Data extraction

Three reviewers performed the search using an African geographic filter comprising country and regional terms forming the filter set, and further (authors: GO, CAB, and TBO) assessed the risk of study bias as data was extracted from each study (S2 File). In case of a disagreement, it was solved through a discussion and consultation with the reviewing supervisor (WZ).

### Publication bias assessment

Forest plots with satisfactory studies included (>10) were generated and we examined publication bias using the funnel plots, Egger's regression test, and Rank Correlation Test. Duval and Tweedie's "trim and fill" method was used for examining the publication bias effect of missing studies on overall estimates [14].

### Statistical analysis

A meta-analysis was conducted for rotavirus infection of proportions in pre and post-vaccine introduction; chi-squared for heterogeneity and; Higgins I-squared (*I*^2^) was used to assess for total heterogeneity/ total variability among studies. For Higgins I-squared (*I*^2^), Figures of over 40% were regarded as an indication of heterogeneity among studies and if the estimated amount of total heterogeneity (Tau *I*^2^) was less than 40%, studies were considered similar. We did not conduct a subgroup analysis because the moderator variables were similar. Meta-regression was also not done because the moderator variables were not continuous. R software version 3.5.1, was used for all the analyses and P-values < .05 were considered statistically significant.

### Quality rating of studies

STROBE Statement for reporting observational studies were devised to portray conclusions about the strength of proof drawn from these studies [15]. A simplified procedure was adopted for rating; in the methods section of the checklist, one point was allocated to each relevant subheading (S2 File). Two independent reviewers authenticated the checklist and went ahead to rate the seventeen observational articles and an agreement was calculated using a weighted kappa statistic. Ten points were the maximum total score for each article. Articles securing 1–4 points were rated poor, 5–7 as fair and 8–10 as good quality articles. The stated objectives of the paper were also matched to the reporting of outcomes within the paper before assigning the final quality.

## Results

**Fig 1:**
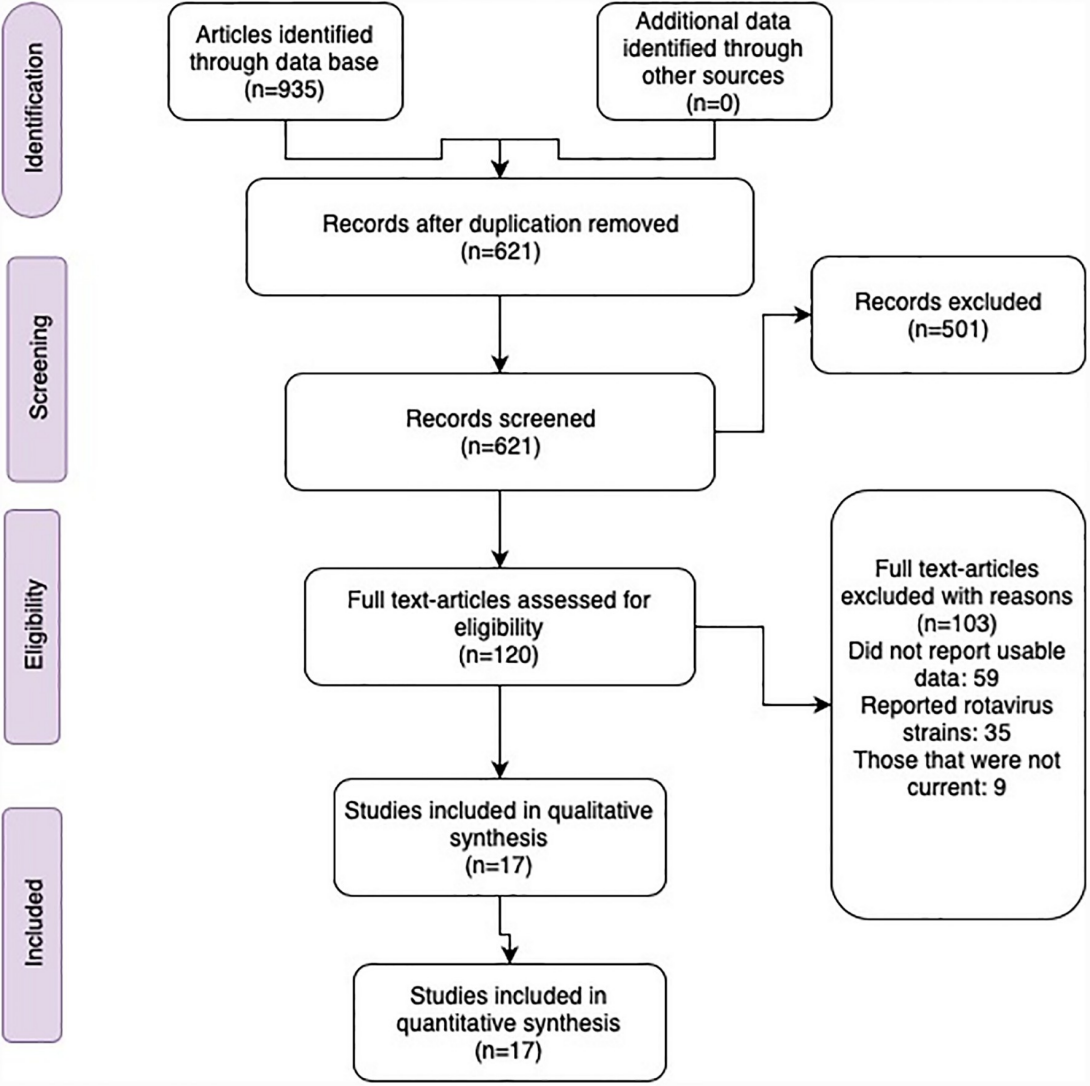
Study profile 2019.

We identified 935 articles. Of these, 621 were unique based on their titles and abstracts, from which 501 were excluded because they did not meet our research objective. The eligibility of 120 full-text articles was assessed. From these 59 did not report usable data (for example, pre and post-vaccination data was not reported), 35 reported rotavirus strain types (rather than endemic surveillance data), 9 were not current, did not distinguish rotavirus from other viruses like norovirus, sapovirus, and did not have sufficient information to meet our inclusion criteria. All the 17 studies reported in this paper were surveillance studies (with prospective follow up), with urban and rural hospitals acting as surveillance sites for collecting samples of the rotavirus cases (Fig 1). Finally, a total of 176698 participants were included in the study (44593 cases out of 132105 tested), and all were children less than five years of age.

### Seasonality and age of RV diarrhea hospitalizations

Hospitalization due to diarrhea among under five years old occurred throughout the year with rotavirus seasonal peaks observed between June-September and was substantially reduced during the post-vaccination period in children <12 months old. However, children aged between 12–24 months demonstrated a slight decline in rotavirus diarrhea hospitalizations [16] (Table 1).

**Table 1:** Study characteristics of 17 studies included for systematic review and meta-analysis by year, country, vaccine, objective, seasonal peak, age group, study type, number of cases and totals tested.

Author	Year	Country	Vaccine	Year introduced	Study objective	Rotavirus Seasonal peaks	Age group	Study type	Number of cases	Total tested
Diop et al	2017	Senegal	RV1	2014/11	Impact of Rotavirus vaccine	No Information	No Information.	surveillance	250	1346
Maphala et al	2017	Swaziland	RV1	2015/05	Impact of Rotavirus vaccine	June-August	No Information.	Surveillance	196	420
Wandera et al	2017	Kenya	RV1	2014/07	Impact of Rotavirus vaccine	June—July	Greater reductions were seen in the second year in the 12–23 months age group.	Surveillance	520	2204
Deus et al	2017	Mozambique	RV1	2015/09	Impact of Rotavirus vaccine	June -September	Reduction in 9–11 month.	surveillance	304	1176
Bonkoungou et al	2017	Burkinafaso	RV5	2013/10	Impact of Rotavirus vaccine		Greater in children 6–11 months of age and reduced in children 12 months.	surveillance	180	571
Rahajamanana et al	2017	Madagascar		2014/05	Impact of Rotavirus vaccine	June- July	No Information.	surveillance	13340	39238
Jani et al	2018	Tanzania	RV1	2013/01	Impact of Rotavirus vaccine	June -September	The reduction was among children 5–23 months.	surveillance	262	868
Sibomana et al	2018	Rwanda	RV5	2012	Impact of Rotavirus vaccine	July- September	Reduced < 5 years.	surveillance	18463	55953
Mpabalwani et al	2018	Zambia	RV1	2012/01	Impact of Rotavirus vaccine	May–July (cool dry months)	In infants (<1 year), there was a median decline in positivity and an increase in positivity in children aged 2–4 years in the pre- and post-vaccine.	surveillance	666	1863
Sanneh et al	2018	Gambia	RV5	2013	Impact of Rotavirus vaccine	January–April	Children age <1 year accounted for 45% of the population infected.	surveillance	133	810
Abebe et al	2018	Ethiopia	RV1	2013/11	Impact of Rotavirus vaccine	July—September	Reduction in children <12 months of age.	surveillance	698	3206
Simwaka et al	2018	Zambia	RV1	2012/01	Impact of Rotavirus vaccine	No Information	No Information.	surveillance	2558	7234
Makaratirwa et al	2018	Zimbabwe	RV1	2014/08	Impact of Rotavirus vaccine	No Information	No Information.	surveillance	3953	10241
Laryea et al	2018	Ghana	RV1	2012/04	Impact of Rotavirus vaccine	No Information	Occurring in children <12 months.	surveillance	461	1080
Bennet et al	2018	Malawi	RV1	2012/10/29	Impact of Rotavirus vaccine	No Information	A decline in children < 11 and 12–23 months showed less substantial decline:	surveillance	403	1135
Tsolenyanu et al	2018	Togo	RV1	2014/06/19	Impact of Rotavirus vaccine	November—January	In the first-year reduction was noted among 1–4-year old’s.	surveillance	843	2034
Lartey et al	2018	Ghana	RV1	2012/04	Impact of Rotavirus vaccine	No Information	No Information	surveillance	1363	2726

RV1; Rotavirus vaccine, Monovalent (Rotarix^®^) and RV5: Rotavirus vaccine, Pentavalent (RotaTeq^®^).

### Proportions of rotavirus infection during pre-vaccination

**Fig 2:**
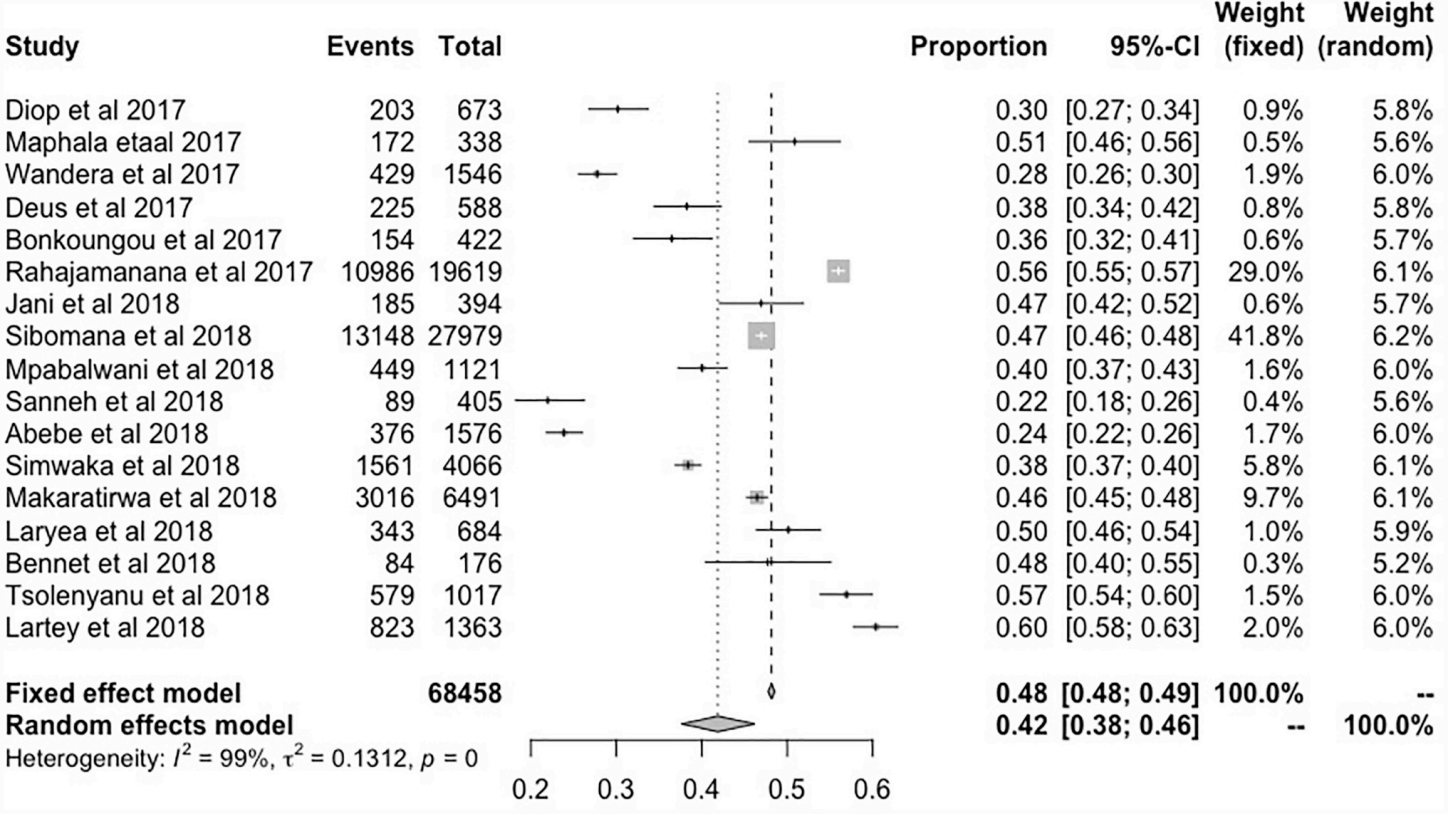
Forest plots showing the proportion of patients tested positive for rotavirus during the pre-vaccination period.

**Fig 3:**
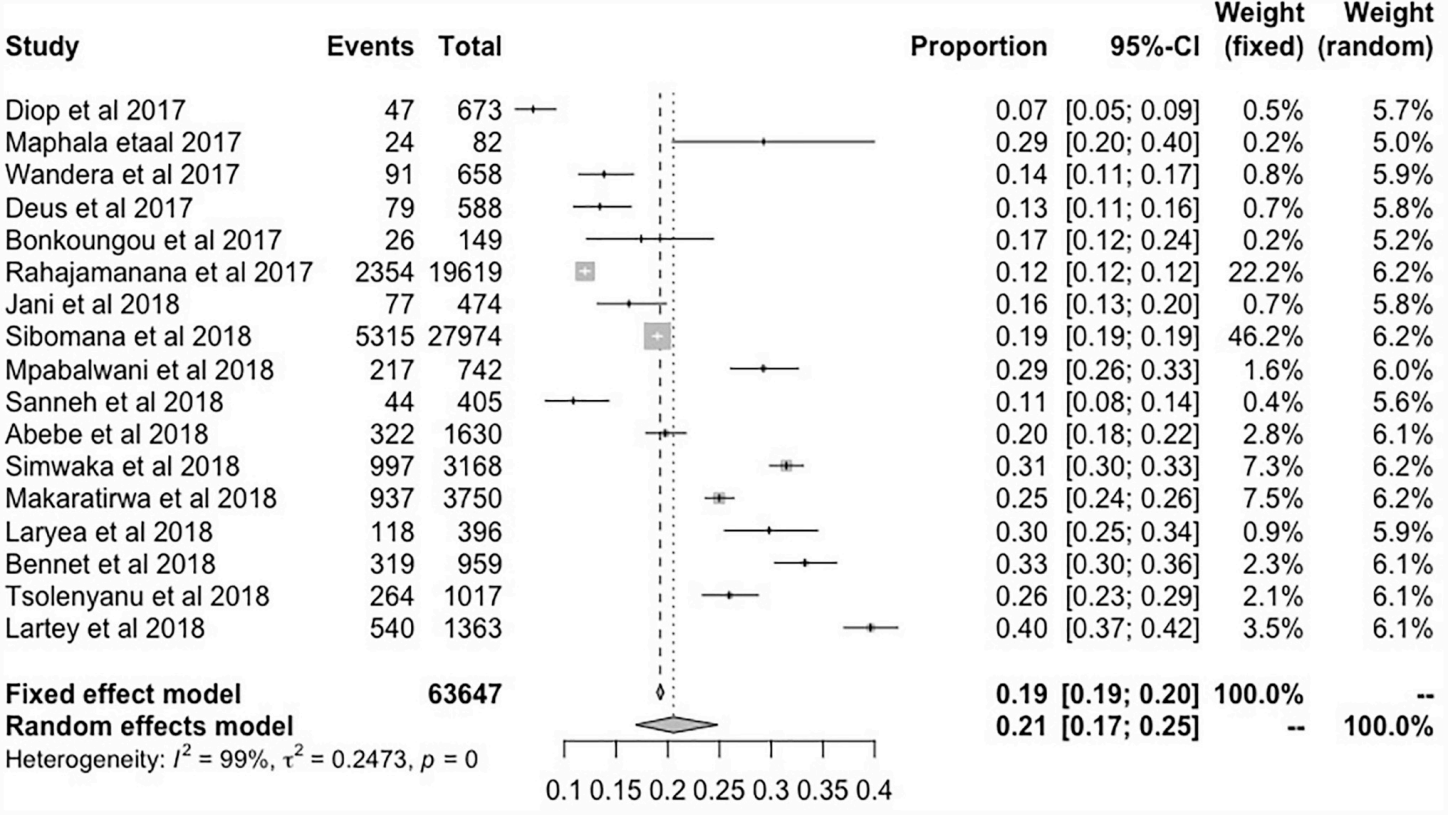
Forest plot showing the proportions of patients tested positive for rotavirus during the post-vaccination period.

**Fig 4:**
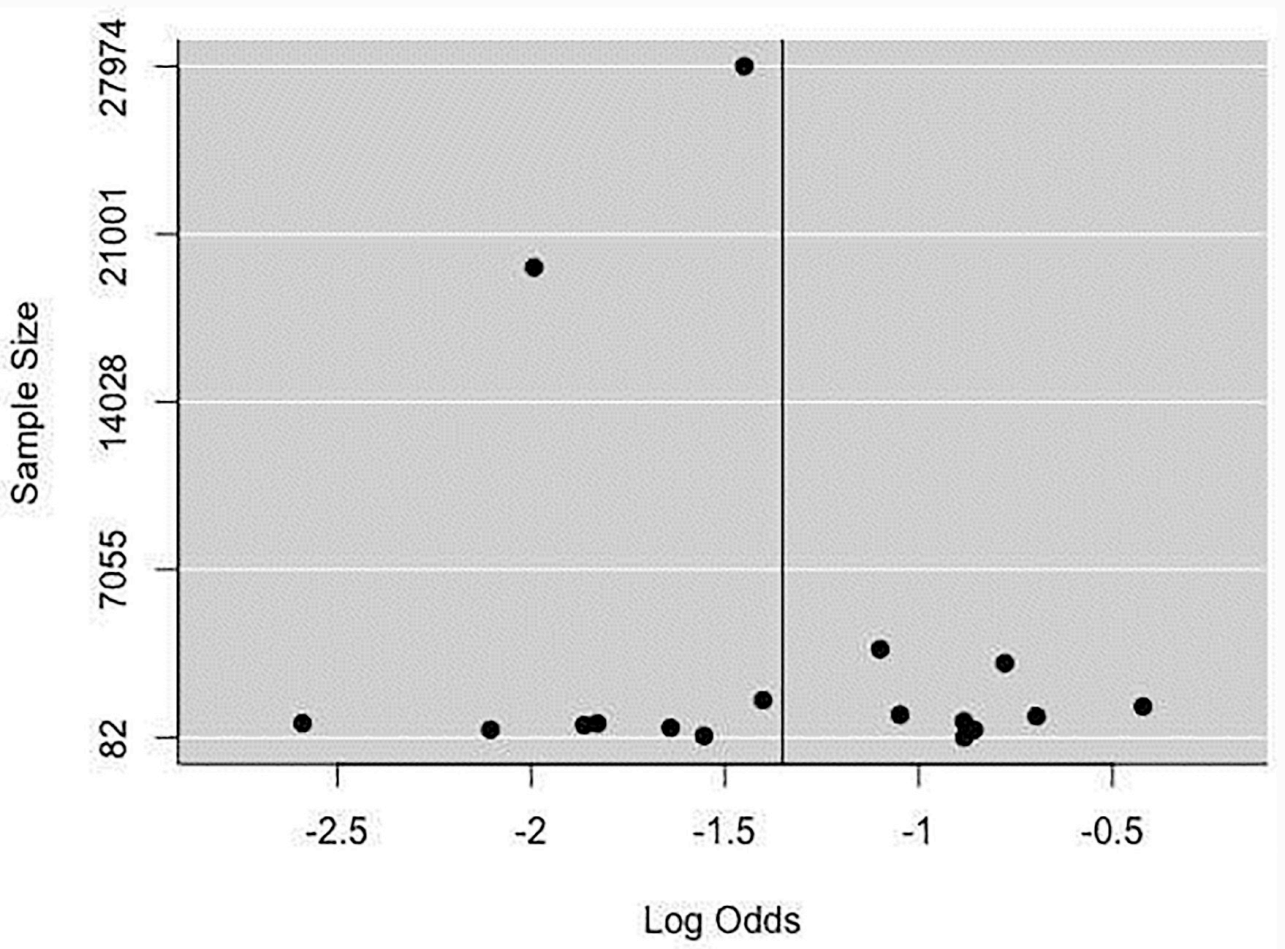
Funnel plot examining publication bias.

**Fig 5:**
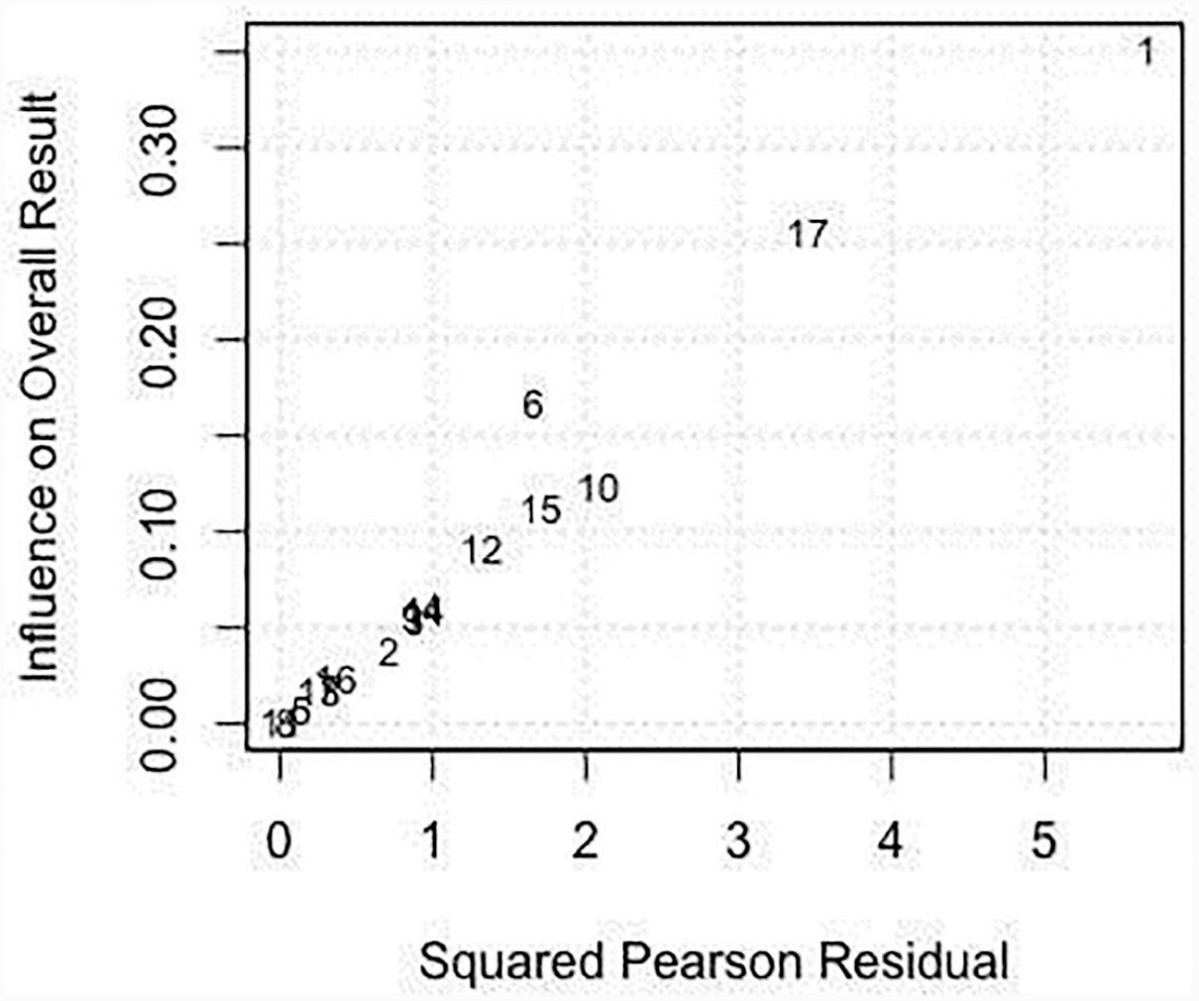
Pearson residual showing outlier studies.

In the 17 studies that were included in the meta-analysis, the summary proportion was presented as a random effect due to heterogeneity of estimates across studies. This was 42% (95% CI: 38–46%), rotavirus positive cases of hospitalized children. The *I*^2^ was 99.00% (95% CI: 99.01–99.77%) of the total variance was between studies. This could have been due to sampling error between studies and other design aspects. Tau *I*^2^ was 13% (95% CI: 0.13–0.57%) (SE = 0.0995). The Q statistic, Q (df = 16) = 1604.6608, p-value<0.001, which indicated that the included studies did shared a common effect size (Fig 2). Three studies (11, 10 and 17) were identified as outliers with a cut off of (> z 2), whereas, the diagnostic test using Baujat plots showed that there was no single study that influenced the results (Fig 3). The Egger's regression test was (z = -1.2642, p = 0.2062), Rank Correlation Test, (Kendall’s tau = 0.0000, p = 1.0000) which indicated that there was no evidence of publication bias (Figs 4 and 5).

### Proportions of rotavirus infection during post-vaccination

**Fig 6:**
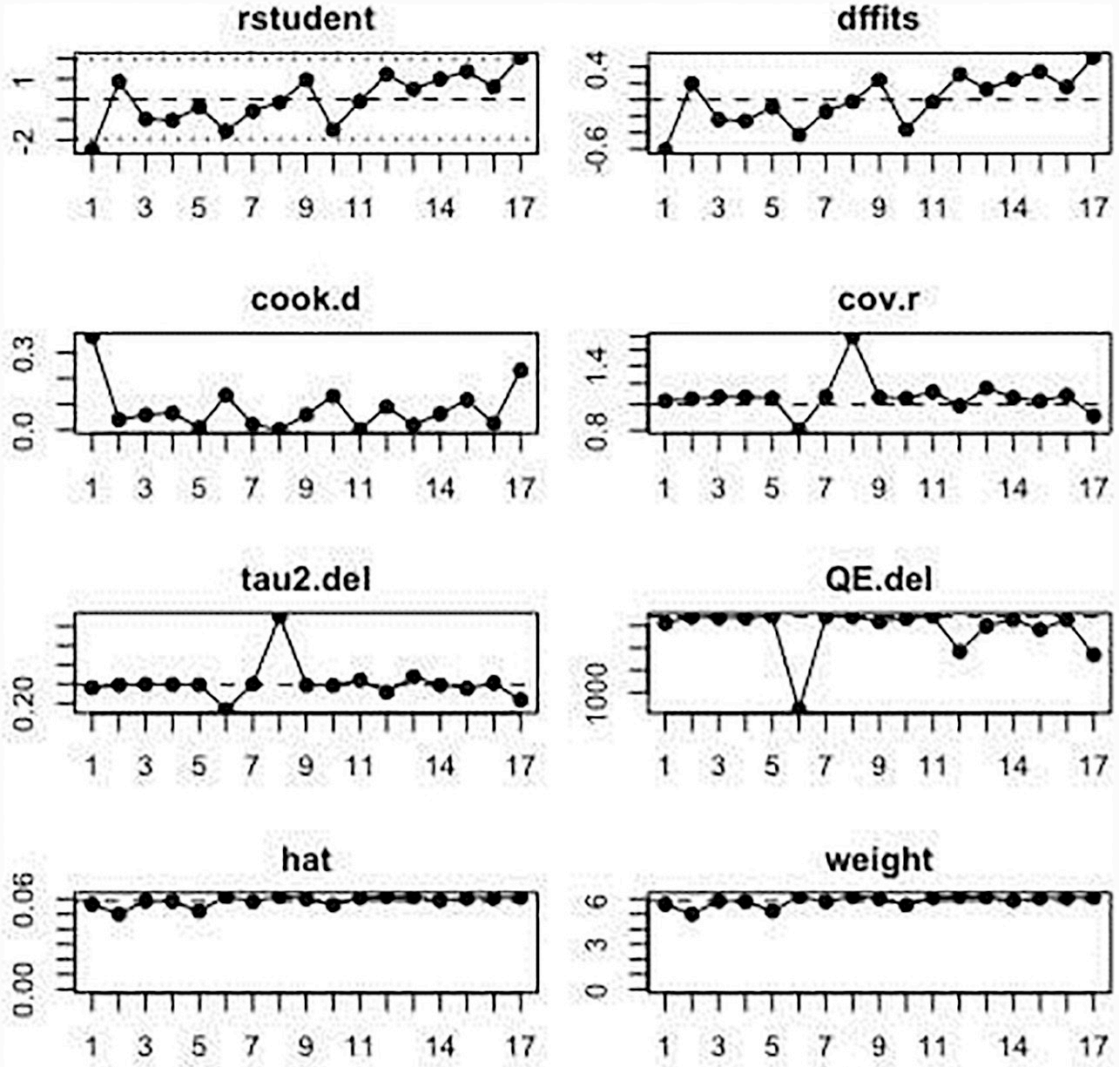
Baujat plot showing no single study that influences the results.

Post-vaccination summary proportion was 21% (95% CI: 17–25%) random effect. The *I*^2^ was 99.05% (95% CI: 98.70–99.70%) as the total variance between studies, possibly this was due to sampling error between studies and other design aspects. However, Tau *I*^2^ was 25% (95% CI: 0.18–0.80%) (SE = 0.1770). The Q statistic, Q (df = 16) = 1682.7045, p-value<0.001, which indicated that the included studies shared a common effect size. So, we concluded that our analysis had substantial homogeneity (Fig 6). Two studies (1, 17) were identified as outliers with a cut off of (> z 2) and the Baujat plot showed that there was no single study that influenced the results (Fig 3). The Egger's regression test was (z = -1.1973, p = 0.2312), Rank Correlation Test, (Kendall’s tau = -0.2794, p = 0.1288) which also indicated that there was no evidence of publication bias (Figs 4 and 5).

## Discussion

This review evaluates the impact of rotavirus vaccine introduction in sub-Saharan Africa countries that met our criteria for inclusion. The review confirms that with the inclusion of rotavirus vaccine (RV1 and RV5) in the respective national immunization programs of African countries, the proportions of rotavirus-positive cases have significantly reduced from 42% (95% CI: 38–46) during the pre-vaccination period to 21% (95% CI: 17–25) post-vaccination period. These findings are consistent with observations in the Caribbean region and Latin America that showed that rotavirus vaccines effectively protect children against diarrhea-related hospitalizations [17]. The vaccine has shown to be highly protective against rotavirus gastroenteritis episodes assessed by a clinical definition for hospitalized cases [18]. In terms of efficacy observed after getting two doses, the rotavirus vaccine is 85% efficient against severe episodes of gastroenteritis and 100 percent efficient against more severe episodes [19].

In Africa, studies have reported reductions in hospitalizations due to diarrhea in general and rotavirus-related infections among children during the post-vaccination period. In South Africa, two studies showed a reduction of 45%-65% [20]. In 2012, it was predicted that rotavirus vaccines would save 53 percent of rotavirus treatment costs and 1,554 lives in Ghana after the introduction of the vaccines [21]. In many parts of the world, studies have shown the impact of rotavirus vaccine on rotavirus-related hospitalizations among children. Our results have also shown that in the first two years post-vaccine execution, the impact was significantly observed, which is comparable to the result in Africa and other parts of the world [22].

In this study, evidence has shown that in children < 12 months of age, the vaccines provided significant protection against hospitalizations caused by rotavirus diarrhea, as compared to in children >12 months. Of course, this could be due to the fading immunity of vaccinated children. These findings are similar to a study conducted in the Caribbean region and Latin America [17]. For infants who were < 1 year of age and eligible for vaccination, there was a significant reduction in the proportions of hospitalization due to acute gastroenteritis during the rotavirus seasonal peaks, implying that vaccination was partly the reason for the decline. The observation implied that rotavirus was the primary cause of diarrhea before the vaccine implementation [23,24]. But also, with the inclusion of rotavirus vaccination in the routine immunization program in Africa, the reduction of hospitalization in children younger than 12 months due to all-cause diarrheas supports the hypothesis.

Previous findings reported during the first post-vaccine seasons have indicated that proportions of rotavirus-positive hospitalizations from other sub-Saharan African countries showed significant decline mostly in infants of 33–51%, but not older children, [24,25]. In low-income and developing countries, children are more likely not to return for the second dose. However, with a single dose of human rotavirus vaccine providing defense against admission to hospital for rotavirus diarrhea, this was quite an encouraging finding because they were protected early in life (>12 months of age) by just a single dose.

### Limitations of the study

The major limitation of this study was that the studies reviewed did not obtain sufficient data on seasonality. The data presented is for only one year of post-vaccine introduction, which may bias our conclusions. Therefore, this limits the interpretation that the possible changes in disease landscape that may be linked to vaccine introduction. Secondly, there may be bias in the analysis of unvaccinated children older than 12–24 months of age from countries that introduced the vaccine later. This may have led to the possibly inaccurate conclusion that rotavirus disease increased in children between 12–24 months of age after rotavirus vaccine introduction. The study participants included were based on hospital surveillance which may not be a good representation for an epidemiological study. Finally, due to the limitations of the data presented in the studies, we were also unable to identify circulating genotypes in the pre-and post-vaccination introduction era. Therefore, a potential in-depth analysis of the dominant circulating genotype is justifiable. Nevertheless, the results of this study have shown that rotavirus associated diarrhea has declined in sub-Saharan Africa.

### Conclusion

Basing on the results from this study, there is evidence that the introduction of the rotavirus vaccine in sub-Saharan Africa, has significantly reduced the burden of rotavirus associated diarrhea by half. Implying that national immunization programs targeting different childhood infectious diseases have led to a decline in disease morbidity. The results also show that there will be a continued decline in the rotavirus disease burden in the years to come. Therefore, with this evidence, there is a need for continued use of the rotavirus vaccines in sub-Saharan Africa. This should also encourage the remaining countries to introduce the vaccine to their national immunization program. This will help protect children from rotavirus associated-diarrheal infections, hospitalizations due to rotavirus-related diarrhea, mortality due to rotavirus diarrhea, most especially during the first year of life.

## Supporting information

S1 File African search filter. (DOCX)

S2 File Quality rating of studies. (DOCX)

S1 Checklist PRISMA checklist. (DOC)
