# Supplementary material for: Evidence of rotavirus vaccine impact in sub-Saharan Africa: Systematic review and meta-analysis
Source: PLoS One. 2020 Apr 27;15(4):e0232113. doi: 10.1371/journal.pone.0232113 (PMC7185587; doi:10.1371/journal.pone.0232113)
Supplement: S1 File — (DOCX) [file pone.0232113.s001.docx]

Appendix: African search filter

(‘‘Africa’’[MeSH] OR Africa*[tw] OR Algeria[tw] OR Angola[tw] OR Benin[tw] OR Botswana[tw] OR ‘‘Burkina Faso’’[tw] OR Burundi[tw] OR Cameroon[tw] OR ‘‘Canary Islands’’[tw] OR ‘‘Cape Verde’’[tw] OR ‘‘Central African Repub- lic’’[tw] OR Chad[tw] OR Comoros[tw] OR Congo[tw] OR ‘‘Democratic Republic of Congo’’[tw] OR Djibouti[tw] OR Egypt[tw] OR ‘‘Equatorial Guinea’’[tw] OR Eritrea[tw] OR Ethiopia[tw] OR Gabon[tw] OR Gambia[tw] OR Ghana[tw] OR Guinea[tw] OR ‘‘Guinea Bissau’’[tw] OR ‘‘Ivory Coast’’[tw] OR ‘‘Cote d’Ivoire’’[tw] OR Jama- hiriya[tw] OR Jamahiryia[tw] OR Kenya[tw] OR Lesotho[tw] OR Liberia[tw] OR Libya[tw] OR Li- bia[tw] OR Madagascar[tw] OR Malawi[tw] OR Mali[tw] OR Mauritania[tw] OR Mauritius[tw] OR Mayote[tw] OR Morocco[tw] OR Mozambique[tw] OR Mocambique[tw] OR Namibia[tw] OR Niger[tw] OR Nigeria[tw] OR Principe[tw] OR Reunion[tw] OR Rwanda[tw] OR ‘‘Sao Tome’’[tw] OR Senegal[tw] OR Seychelles[tw] OR ‘‘Sierra Leone’’[tw] OR Somalia[tw] OR ‘‘South Afri- ca’’[tw] OR ‘‘St Helena’’[tw] OR Sudan[tw] OR Swaziland[tw] OR Tanzania[tw] OR Togo[tw] OR Tunisia[tw] OR Uganda[tw] OR ‘‘Western Sahar- a’’[tw] OR Zaire[tw] OR Zambia[tw] OR Zimba- bwe[tw] OR ‘‘Central Africa’’[tw] OR ‘‘Central African’’[tw] OR ‘‘West Africa’’[tw] OR ‘‘West African’’[tw] OR ‘‘Western Africa’’[tw] OR ‘‘Wes- tern African’’[tw] OR ‘‘East Africa’’[tw] OR ‘‘East African’’[tw] OR ‘‘Eastern Africa’’[tw] OR ‘‘East- ern African’’[tw] OR ‘‘North Africa’’[tw] OR ‘‘North African’’[tw] OR ‘‘Northern Africa’’[tw] OR ‘‘Northern African’’[tw] OR ‘‘South Afri- can’’[tw] OR ‘‘Southern Africa’’[tw] OR ‘‘South- ern African’’[tw] OR ‘‘sub Saharan Africa’’[tw] OR ‘‘sub Saharan African’’[tw] OR ‘‘subSaharan Africa’’[tw] OR ‘‘subSaharan African’’[tw]) NOT (‘‘guinea pig’’[tw] OR ‘‘guinea pigs’’[tw] OR ‘‘aspergillus niger’’[tw])
