# Supplementary material for: Evidence of rotavirus vaccine impact in sub-Saharan Africa: Systematic review and meta-analysis
Source: PLoS One. 2020 Apr 27;15(4):e0232113. doi: 10.1371/journal.pone.0232113 (PMC7185587; doi:10.1371/journal.pone.0232113)
Supplement: S2 File — (DOCX) [file pone.0232113.s002.docx]

Table. Quality evaluation of the 17 studies included in the meta-analysis.

| Study code | ① | ② | ③ | ④ | ⑤ | ⑥ | ⑦ | ⑧ | ⑨ | ⑩ | ⑪ |
| --- | --- | --- | --- | --- | --- | --- | --- | --- | --- | --- | --- |
| 1 | YES | YES | NO | U | NO | YES | NO | YES | NO | NO | - |
| 2 | YES | YES | YES | YES | NO | YES | YES | YES | NO | YES | - |
| 3 | YES | YES | YES | YES | NO | YES | YES | YES | NO | NO | - |
| 4 | YES | YES | YES | YES | YES | YES | NO | YES | NO | YES | - |
| 5 | YES | YES | YES | NO | NO | YES | YES | YES | NO | YES | - |
| 6 | YES | YES | YES | YES | NO | U | NO | YES | NO | NO | - |
| 7 | YES | YES | YES | YES | NO | NO | NO | YES | NO | YES | - |
| 8 | YES | YES | YES | YES | YES | NO | YES | NO | YES | NO | - |
| 9 | YES | YES | YES | NO | NO | NO | YES | NO | NO | YES | - |
| 10 | YES | YES | YES | YES | NO | YES | NO | YES | NO | NO | - |
| 11 | YES | YES | YES | YES | NO | YES | NO | YES | NO | YES | - |
| 12 | YES | YES | YES | YES | YES | YES | NO | YES | YES | YES | - |
| 13 | YES | YES | YES | YES | NO | YES | YES | YES | NO | YES | - |
| 14 | YES | YES | YES | YES | NO | YES | YES | YES | YES | YES | - |
| 15 | YES | YES | YES | YES | YES | YES | YES | YES | NO | YES | - |
| 16 | YES | YES | YES | YES | NO | YES | YES | YES | U | YES |  |
| 17 | YES | YES | U | U | NO | YES | NO | YES | YES | NO |  |

Note: Y, yes; N, no; U, unclear. Items 11 was not appropriate for the studies included in the meta-analysis.

ARHQ Methodology Checklist for Cross-Sectional/Prevalence Study

Website: http://www.ncbi.nlm.nih.gov/books/NBK35156/

| Item | Yes | No | Unclear |
| --- | --- | --- | --- |
| ① Define the source of information (survey, record, review) |  |  |  |
| ②List inclusion and exclusion criteria for exposed and unexposed subjects (cases and controls) or refer to previous publications |  |  |  |
| ③ Indicate time period used for identifying patients |  |  |  |
| ④Indicate whether subjects were consecutive if not population-based |  |  |  |
| ⑤ Indicate if evaluators of subjective components of study were blind to other aspects of the status of the participants |  |  |  |
| ⑥ Describe any assessments undertaken for quality assurance purposes (e.g., test/retest of primary outcome measurements) |  |  |  |
| ⑦ Explain any exclusions of data from analysis |  |  |  |
| ⑧ Describe how confounding was assessed and/or controlled |  |  |  |
| ⑨ If applicable, explain how missing data were handled in the analysis |  |  |  |
| ⑩ Summarize patient response rates and completeness of data collection |  |  |  |
| ⑪ Clarify what follow up, if any, was expected and the percentage of patients for which incomplete data or follow-up was obtained |  |  |  |
